# Essential components of pre-electroconvulsive therapy assessment

**DOI:** 10.1192/j.eurpsy.2024.70

**Published:** 2024-08-27

**Authors:** S. Medved

**Affiliations:** Department of Psychiatry and Psychological Medicine, University Hospital Centre Zagreb, Zagreb, Croatia

## Abstract

**Abstract:**

A thorough pre-electroconvulsive therapy (ECT) assessment is a integral to ECT preparation. Usually, the assessment encompasses elements such as medical history, cognitive assessment, laboratory tests, imaging diagnostics, and consultation with an anesthesiologist. However, there is currently no universally standardized minimal or optimal pre-ECT evaluation at the international level. Recent results show a high variability of the pre-ECT evaluation practice across Europe. Establishing a standardized approach to pre-ECT evaluation would be of great interest for both patients and practitioners.

**Disclosure of Interest:**

None Declared

